# Identification of one B-cell epitope from NS1 protein of duck Tembusu virus with monoclonal antibodies

**DOI:** 10.1371/journal.pone.0181177

**Published:** 2017-07-26

**Authors:** Jinfeng Ti, Zhijie Li, Xiuli Li, Yunjian Lu, Youxiang Diao, Fang Li

**Affiliations:** 1 Zoology Institute, Shan Dong Agricultural University, Shan Dong province, Tai’an, China; 2 Shandong Vocational Animal Science and Veterinary College, Shan Dong province, Weifang, China; Sun Yat-Sen University, CHINA

## Abstract

This study describes the identification of one linear B-cell epitope on TMUV NS1 protein with monoclonal antibody (mAb) 3G2 by indirect enzyme-linked immunosorbent assay (ELISA). In this study, NS1 protein was expressed in prokaryotic expression system and purified. One mAb against NS1 protein was generated from Balb/c mice immunized with recombinant protein NS1. A set of 35 partially-overlapping polypeptides covering the entire NS1 protein was expressed with PGEX-6P-1 vector and screened with mAb 3G2. One polypeptide against the mAb was acquired and identified by indirect ELISA and western-blot. To map the epitope accurately, one or two amino acid residues were removed from the carboxy and amino terminal of polypeptide sequentially. A series of truncated oligopeptides were expressed and purified. The minimal determinant of the linear B cell epitope was recognized and identified with mAb 3G2. The accurate linear B-cell epitope was ^269^DEKEIV^274^ located in NS1 protein. Furthermore, sequence alignment showed that the epitope was highly conserved and specific among TMUV strains and other flavivirus respectively. The linear B-cell epitope of TMUV NS1 protein could benefit the development of new vaccines and diagnostic assays.

## Introduction

Tembusu virus infection in ducks is caused by Tembusu virus (TMUV), which was first reported in April 2010 in China[1]. The virus can infect more varieties of ducks such as Beijing duck, golden duck, Shaoxing duck, Cherry Valley, Campbell ducks, Jinyun duck, etc. Laying ducks infected mainly demonstrated drops in egg production, follicular rupture and bleeding, and yolk peritonitis. Ducklings mainly displayed standing instability and paralysis, retarded growth, with 10 to 30% mortality rates [2, 3]. Other birds such as Chickens, geese, sparrows, etc were also infected and displayed obvious clinical signs [4–6]. So far, the disease has resulted in a great economic loss to the poultry industry and caused wide public concern. There is no specific treatment available for TMUV and the vaccination is an effective way to prevent TMUV infection in waterfowl. The inactivated vaccines and live attenuated vaccines against TMUV have been successfully developed and already used in clinical production[7, 8]. But the live attenuated vaccines could display disadvantages of reversion of virulence and spread, and the inactivated vaccines didn’t display an effective cellular immunity. So the development of a new type of vaccine is very urgent.

TMUV is a mosquito-borne flavivirus which belongs to the Ntaya virus group within flavivirus genus, flaviviridae family [9]. TMUV genome encodes a single polyprotein which is cleaved into three structural proteins (C, prM and E) and seven non-structural proteins (NS1, NS2A, NS2B, NS3, NS4A, NS4B and NS5) [3]. Among them, NS1 protein is a glycoprotein which is present in cell-surface or in the form of cell-associated protein [10]. NS1 protein possesses several unusual and interesting performances which is closely related to the membrane function and indispensable in the early viral replication, assembly and release of the virus [11]. NS1 protein contains multiple protective T-cell and B-cell epitopes which can induce both humoral and cell-mediated immunity without the risk of antibody-dependent enhancement [12, 13]. The B-cell epitopes mainly refer to multiple continuous amino acid residues on the protein surface or the spatial conformation of discontinuous amino acid residues. Accurate analysis of the epitopes on NS1 protein is critical for further understanding the mechanism of NS1-mediated immune protection. In recent years, epitope-based marker vaccines have increasingly attracted wide attentions in public [14]. The identification of linear epitopes on NS1 protein would be conducive to the development of epitope-based marker and subunit vaccines, preparation of protective antibodies and understanding protein functions [15].

It has been demonstrated that NS1 protein of flavivirus has more virus-specific epitopes than cross-reactive ones in contrast to E protein which has more cross-reactive than specific epitopes [16]. Therefore, it is very interesting and valuable to screen and identify epitopes on NS1 protein for specific serological diagnosis that can differentiate between infections caused by flaviviruses [16–18]. In this study, one linear B-cell epitope was identified and characterized with one monoclonal antibody against TMUV NS1 protein. This study can lay the foundation for comprehending the antigenic structure of TMUV NS1 protein, development of a specific serological diagnostic assay and a new type of vaccine for TMUV infection.

## Materials and methods

### Ethics statement

This research was approved by the Committee on the Ethics of Animal of Shandong (permit number 20147620).

### Cell lines

Myeloma cell line SP2/0 and baby hamster kidney BHK-21 cells (CCL-10, American Type Culture Collection) were cultured in DMEM/High glucose (Hyclone, Thermo scientific, USA) in a humidified 5% CO_2_ atmosphere at 37°C. All culture media were added with 10% inactivated fetal bovine serum (TransGen, Beijing, China), 100 U/mL penicillin and 100 mg/mL streptomycin (Solarbio, Beijing, China).

### Virus and serum

The SDSG strain (Accession number: KJ740747.1) was isolated from a duck farm in Shandong province in 2013. The virus titer was 10^4.8^ ELD50/ 0.2mL (Median embryo lethal dose), calculated according to the Reed and Muench method (Reed and Muench, 1938). The duck and mouse sera against TMUV were also provided by Dr. Ke-Xiang Yu.

### Expression of TMUV NS1 gene

The NS1 gene was amplified by conventional PCR using a duck TMUV cDNA template. After agarose gel purification and restriction enzyme digestion, the recombinant plasmid pET-28 -NS1 was constructed and identified. The recombinant plasmid pET-28-NS1 and the control plasmid pET-28a(+) were transformed into BL21 (DE3) cells and induced by isopropyl-thiogalac-topyranoside (IPTG) with the final concentration of 1mmoL/L at 37°C for 1~6h. After induction, bacterial cells were centrifugated and washed with phosphate buffered saline (PBS, pH 7.4). Bacterial cells after washing were lysed by sonication. The recombinant protein NS1 was identified with sodium dodecyl sulfate-polyacrylamide gel electrophoresis (SDS-PAGE) and western-blot. And then it was purified by washing with urea solutions of different concentrations (0.5M, 1M and 3M) and the concentration was measured using a BCA Protein Assay Kit (Kang Wei, China). Finally, the protein was stored in -80°C.

### Production of monoclonal antibodies (mAbs) against TMUV NS1 protein

6-week-old Balb/c mice were purchased from Experimental Animal Center of Shandong Province and housed in SPF isolators that were ventilated under negative pressure. Feed and water were provided ad libitum. 6-week-old Balb/c mice were immunized with the purified NS1 protein and Freund’s adjuvant (Sigma, St. Louis, MO, USA) for three times. Three days after the final immunization, the Balb/c mice were euthanized by carbon dioxide and spleen cells were harvested. Subsequently, mouse splenocytes were fused with sp2/0 murine myeloma cells using 50% polyethylene glycol (PEG 4000, Sigma) according to the procedure described previously[19]. The fusion cells were separated into ninety six well plates and cultured selectively in DMEM/high glucose containing 10% fetal bovine serum, 100 mM hypoxanthine, 16 mM thymidine and 400 mM aminopterin. On day five, 50μL of HAT medium was added additionally in each cell of the 96 cell plates. On day twelve, HAT medium was replaced with fresh HT medium completely. After HAT/HT medium screening, culture supernatants were analyzed by the indirect enzyme-linked immunosorbent assay (ELISA), western blotting, IFA and Blocking ELISA. The positive hybridoma cells were subcloned repeatedly by the limiting dilution method till monoclonal hybridoma cells were obtained. Positive hybridoma cells were cultured in the abdominal cavity of paraffin-primed Balb/c mice to obtain ascitic fluid. The characteristics of monoclonal antibodies were identified using Pierce Rapid Isotyping Kit (Thermo, Rockford, IL, USA). The cell supernatants and ascitic fluids were respectively diluted from 1:10 to 1:20480 by double-dilution method. The titers of diluted antibodies were measured by indirect ELISA.

### Expression of polypeptide proteins

To screen the epitopes of monoclonal antibodies, 35 pairs of primers about 16-amino acid peptides partially-overlapping covering the entire NS1 gene were designed (Table 1). After the forward and reverse primers annealing, the genes encoding 35 pairs of 16-amino acid peptides were amplified. 35 polypeptide fragments were cloned into the pGEX-6p-1 plasmid respectively, identified by sequencing and expressed as fusion proteins with GST as described previously **[20]**. The fusion proteins were identified by SDS-PAGE and purified by Pierce GST Protein Interaction Pull-Down Kit according to the manufacturer’s instructions (Thermo, USA). The concentrations of purified proteins were measured by a BSA Protein Assay Kit (Kang Wei, China).

**Table 1:** Sequences of the overlapping polypeptides from TMUV NS1 (SDSG strain, Accession number: KJ740747.1).

Peptide no.	Amino acid sequence	Peptide no.	Amino acid sequence
NS1-1	^1^DTGCSIDLARKELKCG^16^	NS1-19	^181^TAVMGTAIKGNRAVHS^196^
NS1-2	^11^KELKCGQGMFVFNDVE^26^	NS1-20	^191^NRAVHSDLSYWIESKN^206^
NS1-3	^21^VFNDVEAWKDNYKYYP^36^	NS1-21	^201^WIESKNNGSWKLERAV^216^
NS1-4	^31^NYKYYPSTPRRLAKIM^46^	NS1-22	^211^KLERAVLGEVKSCTWP^226^
NS1-5	^41^RLAKIMVEAHEAGICG^56^	NS1-23	^221^KSCTWPETHTLWSDSV^236^
NS1-6	^51^EAGICGIRSVSRLEHN^66^	NS1-24	^231^LWSDSVVESELIIPKT^246^
NS1-7	^61^SRLEHNMWISIKHELN^76^	NS1-25	^241^LIIPKTLGGPKSHHNT^256^
NS1-8	^71^IKHELNAILEDNAIDL^86^	NS1-26	^251^KSHHNTRTGYKVQSSG^266^
NS1-9	^81^DNAIDLTVVVEENPGR^96^	NS1-27	^261^KVQSSGPWDEKEIVID^276^
NS1-10	^91^EENPGRYRKTNQRLPN^106^	NS1-28	^271^KEIVIDFDYCPGTTVT^286^
NS1-11	^101^NQRLPNVDGELMYGWK^116^	NS1-29	^281^PGTTVTVTSSCRDRGP^296^
NS1-12	^111^LMYGWKKWGKSIFSSP^126^	NS1-30	^291^CRDRGPSARTTTASGK^306^
NS1-13	^121^SIFSSPKMSNNTFVID^136^	NS1-31	^301^TTASGKLITDWCCRSC^316^
NS1-14	^131^NTFVIDGPKTKECPDE^146^	NS1-32	^311^WCCRSCTTPPLRFVTK^326^
NS1-15	^141^KECPDERRAWNSMKIE^156^	NS1-33	^321^WCCXXCTTPPXXFVTK^336^
NS1-16	^151^NSMKIEDFGFGVLSTK^166^	NS1-34	^331^YGMEIRPIAHGDDML^346^
NS1-17	^161^GVLSTKVWMEMRTENT^176^	NS1-35	^341^GDDMLIKSKVMA^352^
NS1-18	^171^MRTENTTDCDTAVMGT^186^		

### Truncated expression of short peptide proteins

In order to precisely locate the exact sequences of linear B-cell epitopes on NS1 protein, one or two amino acid residues were removed from the carboxy terminal of the peptide sequentially (Table 2). Ten pairs of primers were designed according to the shortened peptide sequences. The primers were annealed and gene sequences of truncated peptides were amplified. Ten oligopeptide fragments were cloned into PGEX-6P-1 vectors respectively and expressed as fusion proteins with GST as previously [20]. The oligopeptide proteins were purified by Pierce GST Protein Interaction Pull-Down Kit according to the manufacturer’s instructions (Thermo, USA). The concentrations of purified proteins were measured by a BSA Protein Assay Kit (Kang Wei, China).

**Table 2:** Sequences of the truncated NS1-27 polypepide.

Peptide no.	Amino acid sequence	Peptide no.	Amino acid sequence
NS1-27-F-2	259QSSGPWDEKEIVID276	NS1-27-F-9	252EKEIVID276
NS1-27-F-4	257SGPWDEKEIVID276	NS1-27-R-2	259KVQSSGPWDEKEIV274
NS1-27-F-6	255PWDEKEIVID276	NS1-27-R-3	259KVQSSGPWDEKEI273
NS1-27-F-7	254WDEKEIVID276	NS1-27-R-4	259KVQSSGPWDEKE272
NS1-27-F-8	253DEKEIVID276	NS1-27-R-6	259KVQSSGPWDE270

### Enzyme-linked immunosorbent assay (ELISA)

Each well of 96-well plates was coated with purified fusion proteins subsequently in 0.1 M carbonate buffer (pH 9.6) and incubated at 4°C overnight. After washing three times with PBST (0.5% Tween-20 in PBS), the plates were blocked with 5% skimmed milk for 2 h at 37°C. After washing, cell supernatants of different hybridomas were added to each well respectively and incubated at 37°C for 1h. After the plates were washed three times with PBST, a goat anti-mouse HRP-conjugated polyclonal serum diluted 1:5000 with 5% skimmed milk (TransGen, FS101-02, Beijing, China) was added and incubated for 1 h at 37°C. After washing three times, TMB was added into the wells as a substrate for HRP and incubated in dark for 15 min. 2M H_2_SO_4_ was used to stop the reaction and the absorbance was recorded at 450 nm by a Microplate Absorbance Reader (BioTec, USA). In the process of screening mAbs against TMUV NS1 protein, 96-well plates were coated with recombinant protein NS1 and BL21 (DE3) cells containing plasmid pET-28a(+) respectively. In the process of screening antigenic epitopes on NS1 of TMUV, 96-well plates were coated with glutathione purified GST-NS1 and S-transferase (GST). Mouse sera against TMUV and murine myeloma cells supernatants were used as positive and negative controls. Results depict the average of duplicate assays.

### Western blotting

The expressed fusion proteins were subjected to gel electrophoresis on 12% SDS-PAGE after denaturation with 2 × SDS loading buffer at 100°C for 3~5 min. And protein bands were transferred to a nitrocellulose membrane. The nitrocellulose membrane was blocked with 5% skimmed milk overnight at 4°C. After washing three times with TBST(20 mM Tris-HCl, 150 mM NaCl, 0.01% Tween-20, pH7.5), the membranes were incubated with duck serum against TMUV or mAb against NS1 protein at 37°C for 1 h. After washing three times with TBST, the membranes were immersed into a goat anti-duck or anti-mouse HRP-conjugated polyclonal serum diluted 1:5000 with 5% skimmed milk (KPL, 04-25-6, USA; TransGen, FS101-02, Beijing, China) for 1 h at 37°C. After washing in the same way, membranes were colored in DAB liquid at dark for 1~3 mins. Running water was used to stop the reaction.

### Immunofluorescence assay (IFA)

BHK-21 cells were separated into a 24 well plate (Costar Corning, Corning, NY) and inoculated with TMUV (10^3.5^ ELD_50_/0.2mL duck embryo allantoic fluid). When 80% Cytopathic effect (CPE) of cells occurred, cells were fixed with methanol and acetone (1:1) for 20 min at -20°C. After washing three times with PBS, the monoclonal antibody against TMUV NS1 protein diluted 1:100 with PBS was added and incubated for 1 h at 37°C. After washing three times with PBS, cells were inoculated with a goat anti-mouse IgG(H+L) with FITC conjugate (TransGen, Beijing, China) for 1 h at 37°C. Finally, the reaction result was observed under the fluorescence microscope (Nikon, Eclipse, TE2000-S, Japan). BHK-21 cells without inoculation with TMUV were used as the control.

### Blocking ELISA

Each well of 96 well plates was coated with purified NS1 protein in 0.1 M carbonate buffer (pH 9.6) and incubated at 4°C overnight. After washing three times with PBST (0.5% Tween-20 in PBS), plates were blocked with 5% skimmed milk for 2 h at 37°C. After washing three times with PBST, mouse positive serum against TMUV NS1 protein was added to wells and incubated at 37°C for 1h. After washing three times with PBST, mAb against TMUV NS1 protein was added to wells and incubated at 37°C for 1h. After plates were washed three times with PBST, a goat anti-mouse HRP-conjugated polyclonal serum diluted 1:5000 with 5% skimmed milk (TransGen, FS101-02, Beijing, China) was added and incubated for 1 h at 37°C. After washing three with PBST, TMB was added into the wells as a substrate for HRP and incubated in dark for 15 min. 2M H_2_SO_4_ was used to stop the reaction and the absorbance was recorded at 450 nm by a Microplate Absorbance Reader (BioTec, USA). Results depict the average of duplicate assays. The adajacent value of blocking ELISA was determined using 25 mouse negative sera.

### Dot-ELISA

The nitrocellulose (Millipore, USA)A was punched a series of small circles by a hole puncher. About 2 μg of each purified fusion protein was spotted onto small circles of the nitrocellulose membrane. After the nitrocellulose membrane was dried, it was blocked with 5% skimmed milk at 37°C for 2h. After washing three times with TBST, the membrane was incubated with different mAbs respectively at 37°C for 1 h. After washing three times with TBST, the membrane was incubated with a goat anti-mouse HRP-conjugated polyclonal serum (TransGen, FS101-02, Beijing, China) at 37°C for 1 h. After washing three times with TBST, membranes were colored in DAB liquid. Running water was used to stop the reaction.

## Results

### Production of MAbs against TMUV NS1protein

**Fig 1:**
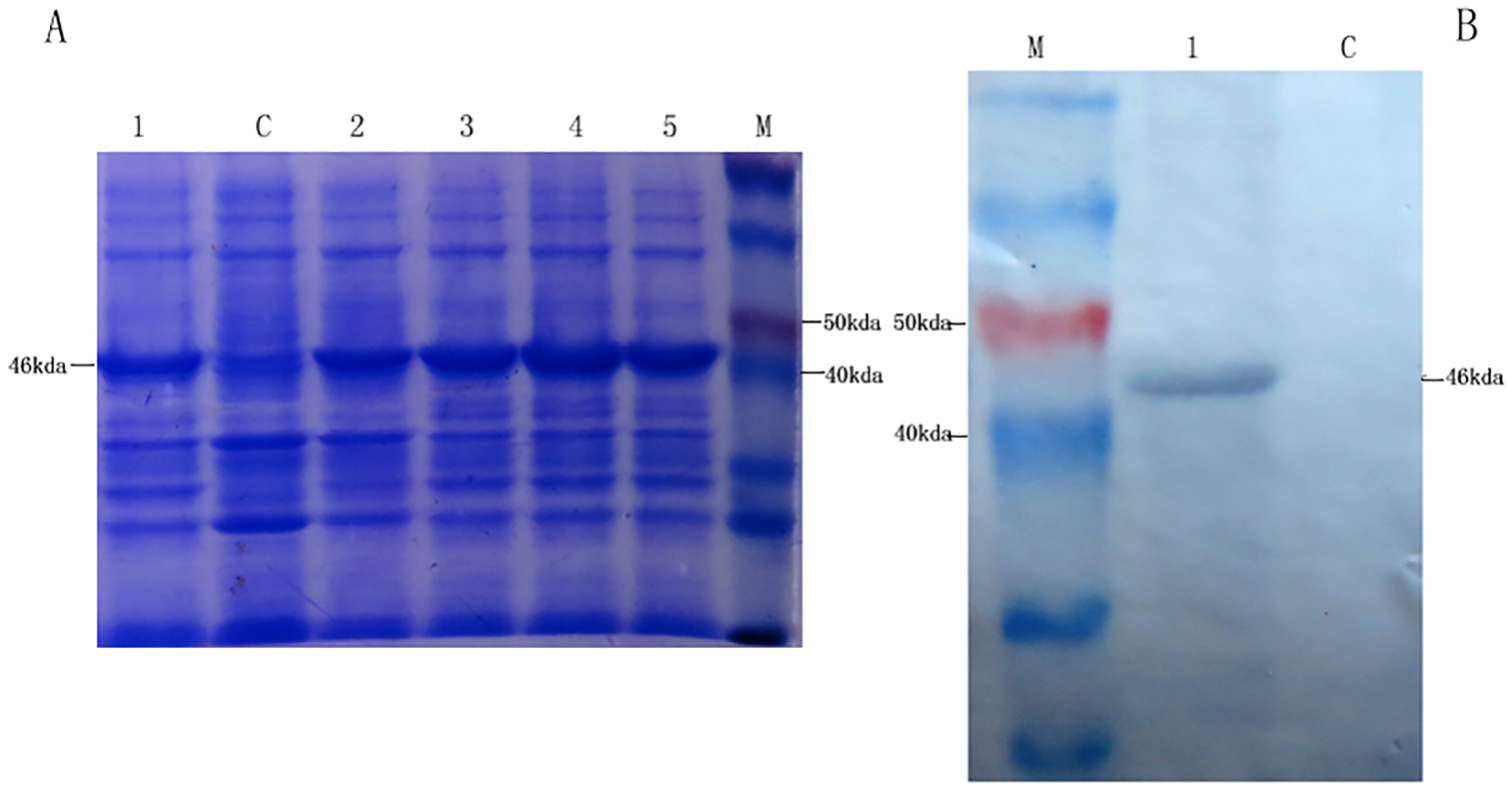
SDS-PAGE and western-blot identification of TMUV NS1 protein. A: SDS-PAGE identification Lane M, Blue PlusIIProtein Marker; Lane 1–5, the bacteria containing PET-NS1 were induced by IPTG for 2h, 3h, 4h, 5h, 6h; Lane C, the bacteria containing PET-28a were induced by IPTG. B: Western-blot identification Lane M, Blue plusIIprotein Marker (14-120kda, Transgen Biotech); Lane 1, The band of NS1-His fusion protein was visualized with TMUV-positive duck serum; Lane 2, Control, His-tag didn’t react with TMUV-positive duck serum.

**Fig 2:**
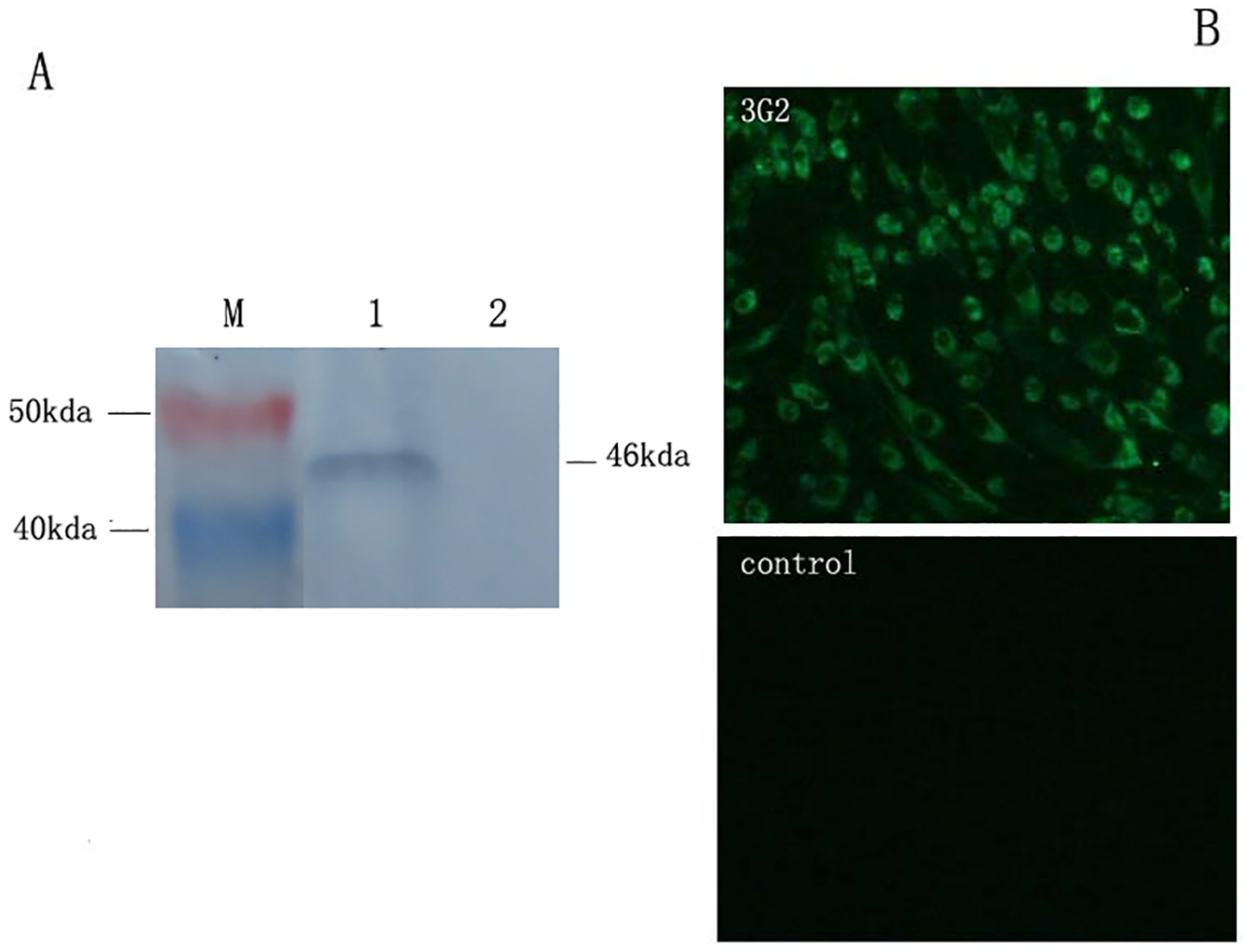
IFA and western-blot identification of mAb 3G2. A: Western-blot identification Lane 1, Control, GST-tag didn’t react with mAb 3G2; Lane 2, The band of NS1-GST fusion protein was reacted with mAb 3G2; Lane M, Blue plusIIprotein Marker (14-120kda, Transgen Biotech). B: IFA identification Monoclonal antibody against TMUV NS1 protein was used to perform IFA on TMUV-infected BHK-21 cells. BHK-21 cells infected with TMUV yielded significant fluorescence with six MAbs in the cytoplasm; Control BHK-21 cells didn’t yield any fluorescence.

Recombinant protein NS1 was successfully expressed in BL21 (DE3) and the highest expression level emerged at 5 hours after induction with 1mM IPTG (Fig 1A). The fusion protein was about 46kda and present in inclusion bodies of bacterial lysates. It could react with the TMUV-positive serum from ducks (Fig 1B). Purified NS1 proteins were used to immunize BALB/c mice. One hybridoma cell line was acquired by subcloning and screening, which produced mAb that could strongly recognize native NS1 protein of TMUV and recombinant NS1 protein by indirect immunofluorescence assay (IFA) (Fig 2) and Western blot (Fig 2). The mAb was named 3G2 which belonged to the subtype of IgG2a and the light chain was kappa. The titers of culture supernatants and ascitic fluids were 1:5120 and 1:10240 measured by indirect ELISA. The adajacent value of Blocking ELISA in this study was 18.67%. The inhibition rate of mouse positive serum against TMUV NS1 protein to mAb 3G2 was 69.34% and much higher than the adajacent value.

### Preliminary analysis of antigenic epitopes

**Fig 3:**
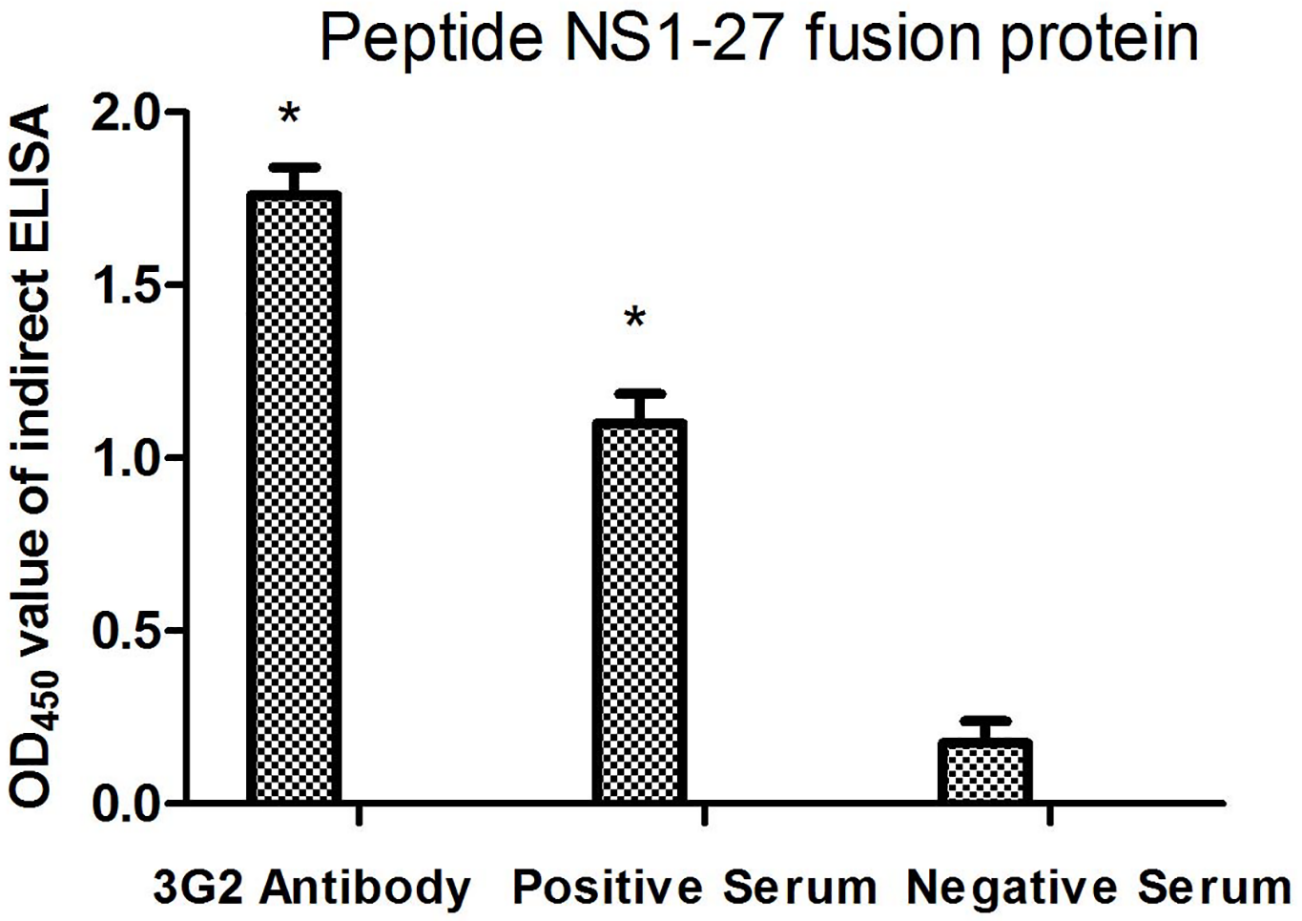
Primary screening of epitope with mAb 3G2. One 16-AA polypeptide of TMUV NS1 protein (NS1-27) was screened with mAb 3G2 by indirect ELISA. Mouse serum against TMUV NS1 protein and normal mouse serum were used as positive and negative controls, respectively. Each sample was detected in triplicate. Error bars were expressed as standard deviation of the means (n = 3). The mean value was statistically significant, calculated by the two-tailed Student’s unpaired t-test (*P < 0.05).

**Fig 4:**
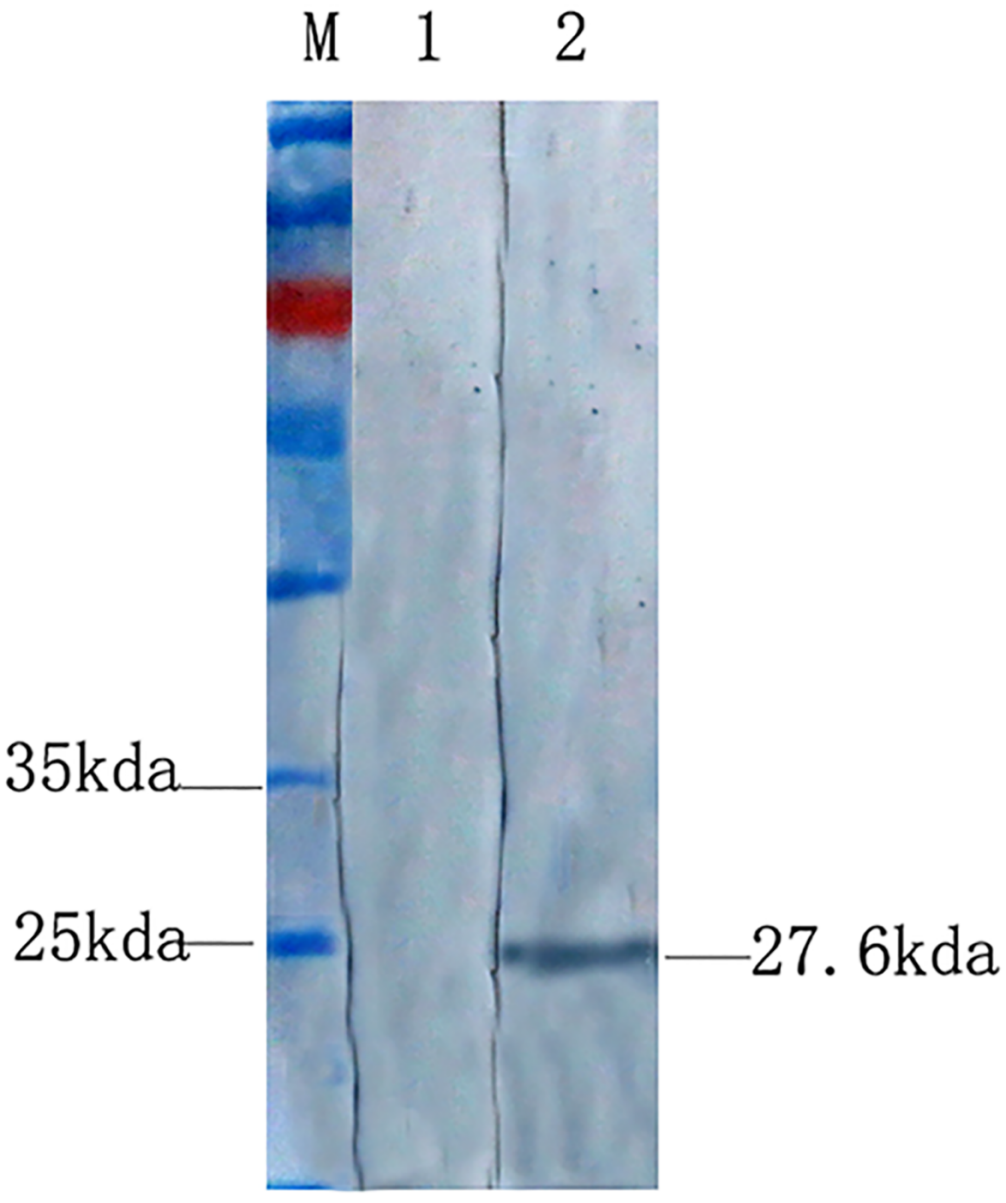
Western-blot identification of epitope. The 16-AA polypeptide of NS1-27 reacted with mAb 3G2 in Western-blot assay. Lane M, PageRuler Prestained Protein Ladder (Fermentas, Canada); Lane 1, GST-tag didn’t react with mAb 3G2; Lane 2, The band of NS1-27-GST fusion protein was visualized with mAb 3G2.

To screen the antigenic epitope of TMUV NS1 mAb, 35 short peptide fusion proteins were successfully expressed in prokaryotic expression system and purified. According to the results of indirect ELISA, one 16 AA peptide protein was recognized by mAb 3G2 (Fig 3). The peptide was NS1-27 which exhibited a positive immunoreactive band of approximate 26 kda with mAb 3G2 by western-blot (Fig 4). No immunoreactivity was detected in the control bands.

### Accurate mapping of the linear epitope

**Fig 5:**
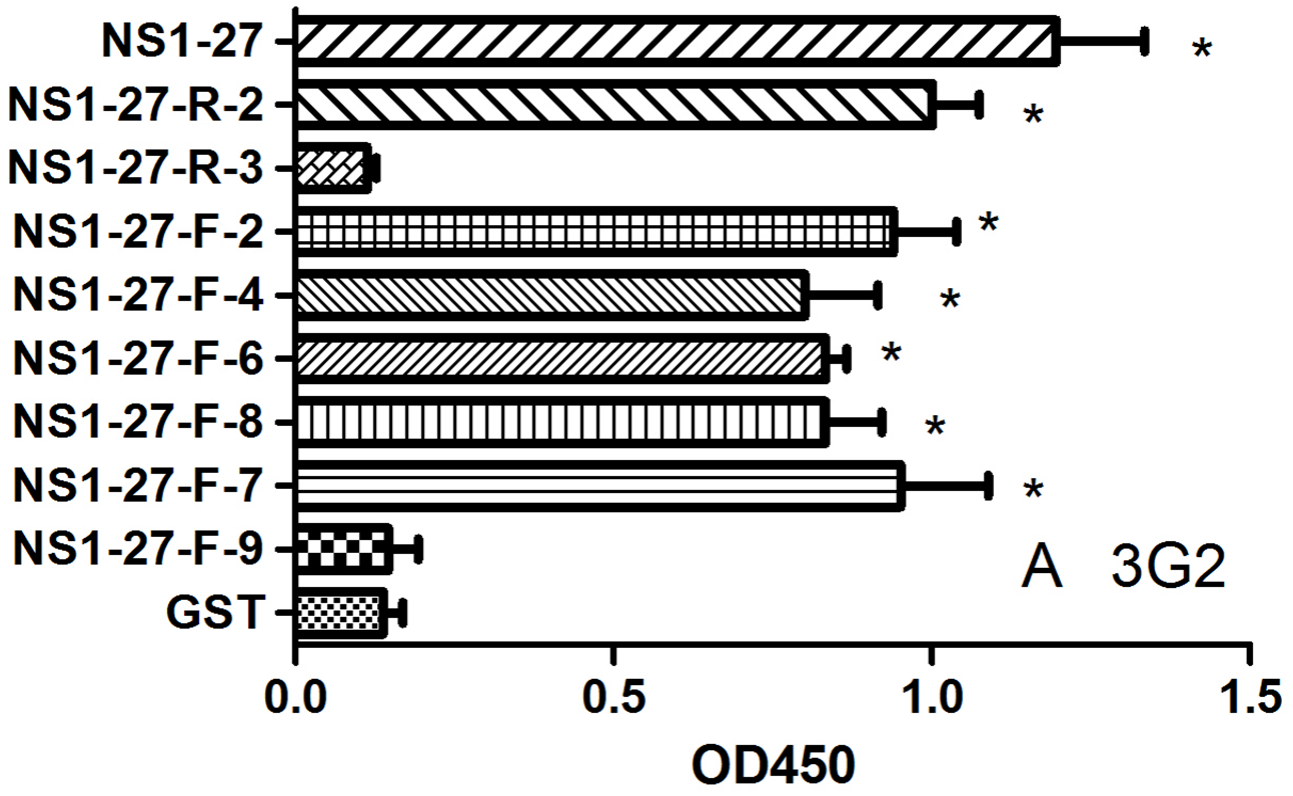
The accurate mapping of one B cell epitope with mAb. NS1-27 polypeptide was truncated from the carboxy and amino terminals. After the truncated peptides were expressed as a GST fusion protein, they were probed with mAb 3G2 by indirect ELISA respectively. The minimal unit of the peptide was the sequence of 8 AA and 3 AA truncated from the carboxy and amino terminals of NS1-27.

To further map the accurate position of the epitope, one or two amino acid residues were cut down from both sides of NS1-27 polypeptide. Ten truncated oligopeptides were expressed in the prokaryotic expression system and purified by different concentrations of urea solution. The truncated fusion peptides were detected using mAb 3G2 by indirect ELISA. The results showed that mAb 3G2 can react with NS1-27-F-2, NS1-27-F-4, NS1-27-F-6, NS1-27-F-7, NS1-27-F-8 and NS1-27-R-2 oligopeptides, while not with NS1-27-F-9, NS1-27-R-3, NS1-27-R-4 and NS1-27-R-6 (Fig 5). So the accurate epitope recognized by mAb 3G2 was the ^269^DEKEIV^274^ sequence which was mapped by truncating 8 AA and 3 AA from 5’ end and 3’ end of NS1-27 polypeptide respectively.

### Identification of accurate linear epitopes

**Fig 6:**
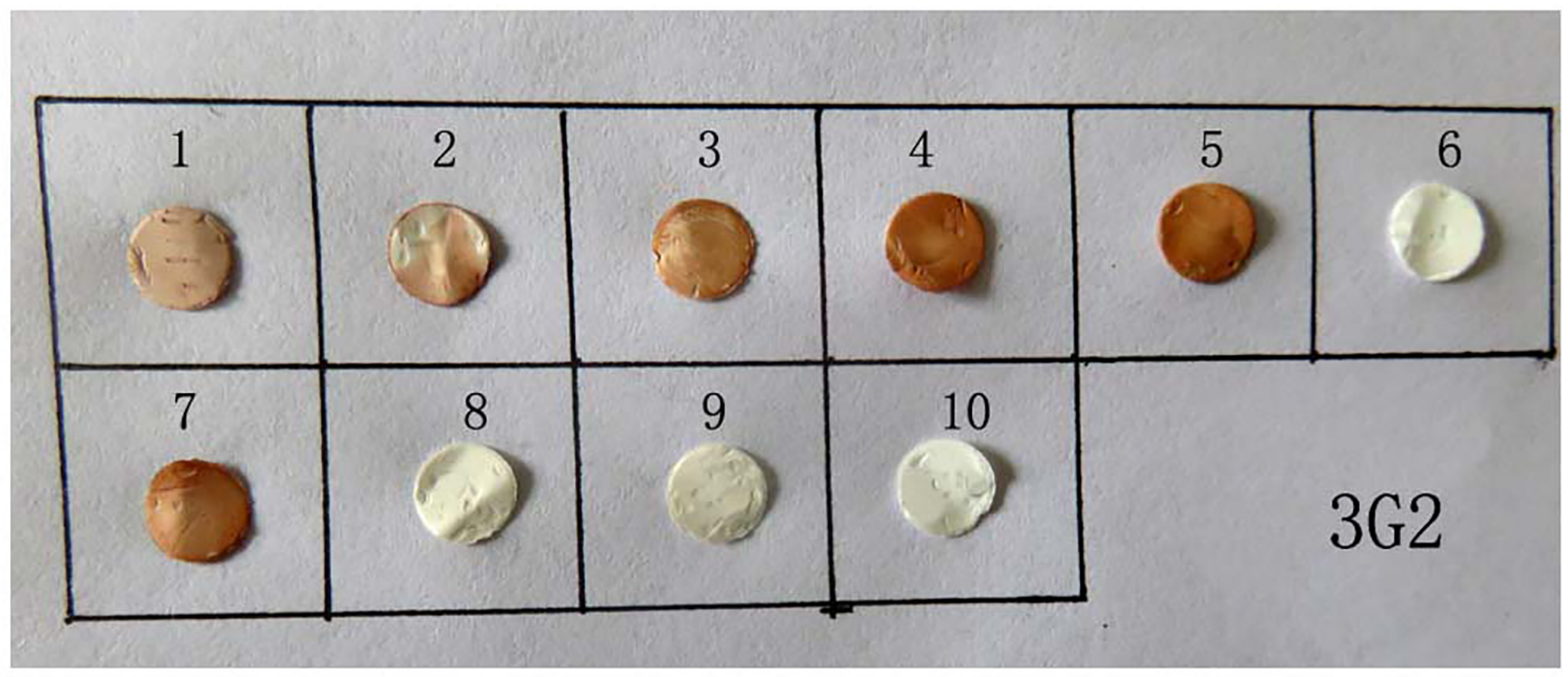
Identification of the accurate epitope with Dot-ELISA. 1–10 small circles of NC were expressed the reaction results of NS1-27-F-2 oligopeptide, NS1-27-F-4 oligopeptide, NS1-27-F-6 oligopeptide, NS1-27-F-7 oligopeptide, NS1-27-F-8 oligopeptide, NS1-27-F-9 oligopeptide, NS1-27-R-2 oligopeptide, NS1-27-R-4 oligopeptide, NS1-27-R-6 oligopeptide, and NS1-27-R-7 oligopeptide with mAb 3G2. Brown NC circles were expressed positive reaction and colorless NC circles were expressed negative reaction.

For the truncated oligopeptides of NS1-27 peptide, Dot-ELISA results showed that nitrocellulose (NC) membranes spotted six oligopeptides of NS1-27-R-2, NS1-27-F-2, NS1-27-F-4, NS1-27-F-6, NS1-27-F-7 and NS1-27-F-8 were colored to red and brown, while NC membranes spotted four oligopeptides of NS1-27-F-9, NS1-27-R-3, NS1-27-R-4, and NS1-27-R-6 were not colored. The results of Dot-ELISA were consistent with the results of indirect ELISA (Fig 6).

### Homology analysis of B-cell epitope on NS1 of TMUV

**Fig 7:**
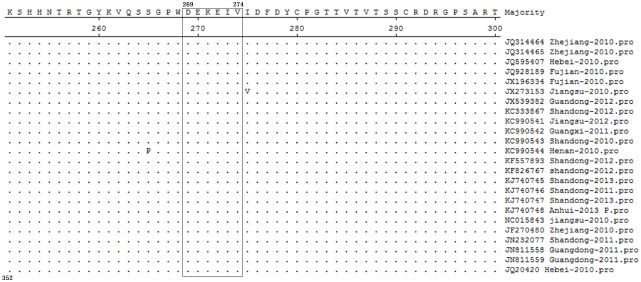
Sequence alignment of 24 TMUV strains around the epitope-coding region of the NS1 protein. Amino acid positions for each individual sequence are numbered on the left. Dots indicate identical amino acids. The identified epitope is in box.

In order to identify conservation of the NS1-27 epitope (^269^DEKEIV^274^), the multiple sequence alignment was performed among 24 different TMUV isolates published in Genbank. The results showed that the B-cell epitope of ^269^DEKEIV^274^ was highly conserved and the amino acid homology was 100% among 24 TMUV strains (Fig 7). In order to identify the specificity of B-cell epitope, the sequence alignment was performed among other flaviviruses such as JEV, DENV, WNV, YFV, BAGV, and MVEV isolates published in Genbank. The results showed that the B-cell epitope of ^269^DEKEIV^274^ had 67% identity with JEV NS1 sequences and 50% identity with BAGV. In addition, the amino acid homology of the epitope was below 50% among other flaviviruses. Especially there was no identity between the epitope and DENV (1–4) NS1 sequences. So NS1-27 epitope had high specificity among other flaviviruses (Table 3) and could be applied to the development of TMUV epitope-based vaccine and serological diagnosis method.

**Table 3:** Sequence alignment of TMUV strains and other flaviviruses around the epitope-coding region of the NS1 protein.

Flavivirus strain	Accession number	Peptide sequence(NS1-27)
byd1	JQ92042	D E K E I V
BAGV	AY632545	- - R D - T
JEV	FJ495189	- - N G - -
MVE	AF161266	- - E D E K
WNV	JF488093	- - G R V E
DENV1	AY762084	H L G K L E
DENV2	EF051521	H L G K L E
DENV3	AY496874	H L G K L E
DENV4	AF161266	H L G K L E
YFV	JX898880	M Q V P L E

Dots indicate identical amino acids.

## Discussion

Tembusu virus infection has been an emerging flavivirus disease in waterfowls in recent years and resulted in severe economic losses to poultry industry. The study of detection, diagnosis, and prevention and control of the disease is a hot issue in the field of Tembusu virus research. NS1 protein is the non-structural protein of flavivirus, which has important functional properties in virus RNA replication, anti-viral immunity and host recognition of virus-associated molecular patterns[11, 21–23]. It is reported that NS1 protein or mAbs against NS1 protein can confer active or passive immunity protection from the flavivirus challenge [24–26], which is closely related to the epitopes on NS1 protein. Therefore, it is important to identify the epitopes on NS1 protein for epitope-based vaccine designment and development of epitope-based serological tests. In our study, one B-cell epitope on TMUV NS1 protein was precisely mapped and analyzed which led to a better understanding of host immune responses and the development of new vaccines and diagnostic tools for TMUV.

MAbs against definite epitopes can provide an important experimental way for studying protein structure, and developing diagnostic and therapeutic reagents for pathogen control [27–29]. Precise analysis of epitopes on flavivirus NS1 protein is very critical for comprehending the mechanism of NS1-mediated immune function. So far, a few reports have described the mapping B-cell epitopes on NS1 protein of JEV[20], WNV[14], DENV[30, 31], and TBEV [32]. And moreover there are also several methods of screening B-cell epitope such as peptide scanning technique, phage peptide library presenting technolog, eukaryotic expression presenting system and prokaryotic expression presenting system. The method of prokaryotic expression system[15, 33] is simple and easy to operate and need not special instruments and technologies. Moreover it is at low cost and common laboratory can afford it in comparison to other methods s. But the method is generally applicable to the screening of linear epitopes and it has extensive workload. Although this method has certain defects, it is very accurate and effective in epitope screening. And so far its application is very broad. In this study, one B-cell epitope was screened by prokaryotic expression system to express a series of small peptides of truncated protein and analyzed by Western Blotting. TMUV NS1-specific mAb was acquired with prokaryotic expression NS1 protein of TMUV. A pane of 16-AA polypeptides of NS1 protein was expressed and one 16-AA polypeptide NS1-27 was screened and identified by 3G2 mAb. In order to accurately map the B-cell epitope, a set of truncated fusion oligopeptides of NS1-27 were expressed. One linear B-cell epitope of ^269^DEKEIV^274^ was confirmed by probing the shortened oligopeptide proteins with 3G2 mAb. In recent years, epitope-based vaccine and specific diagnostic tools have received extensive attentions. The B-cell epitope of TMUV NS1 protein could apply into the development of detection methods to investigate whether the detected antibody was a result of inactivated vaccine immunization or live virus infection.

Sequence alignment showed that NS1-27 epitope of ^269^DEKEIV^274^ was highly conserved among TMUV strains. And furthermore, NS1-27 epitope had 100% identity among 24 TMUV strains. Compared with other flavivirus, the epitope had less 50% identity than DENV (1–4), YFV, WNV, and MVE excluding JEV and BAGV. Moreover, JEV and BAGV couldn’t infect poultry. So the highly conserved and specific nature of the identified epitope is beneficial for the application in vaccine design and epitope-based diagnosis.

In summary, one highly conserved B-cell epitope on TMUV NS1 protein were precisely screened and identified which could provide an important basis and data for understanding the antigenic structure of NS1 protein and the clinical application of epitope-mediated detecting and diagnostic methods.
